# 毛细管电泳-质谱技术在手性化合物分离分析中的研究进展

**DOI:** 10.3724/SP.J.1123.2021.11006

**Published:** 2022-06-08

**Authors:** Zhongmei CHI, Li YANG

**Affiliations:** 1.渤海大学化学与材料工程学院, 辽宁 锦州 121013; 1. College of Chemistry and Materials Engineering, Bohai University, Jinzhou 121013, China; 2.东北师范大学化学学院, 吉林 长春 130024; 2. Faculty of Chemistry, Northeast Normal University, Changchun 130024, China

**Keywords:** 毛细管电泳, 质谱, 手性化合物, 综述, capillary electrophoresis (CE), mass spectrometry (MS), chiral compounds, review

## Abstract

目前使用的绝大多数药物为手性化合物,它们具有相似的物理和化学性质,但药理活性不同,且常以外消旋混合物的形式存在,因此对手性化合物的分离在生物、环境、食品和医药等领域一直备受关注。与广泛使用的液相色谱-质谱(LC-MS)相比,毛细管电泳-质谱(CE-MS)作为一种新型分离分析技术,具有分离效率高、样品和试剂消耗量低、选择性高和分离模式多样化等诸多优势,已经发展成为手性分析领域中有广阔应用前景的分析方法之一。CE-MS结合了CE的高分离效率和低样品消耗以及MS的高灵敏度和强结构解析能力,在蛋白质组学和代谢组学等领域发挥了重要作用。CE杰出的手性拆分能力与MS优势的结合,亦使CE-MS成为实现手性化合物高效分离分析的完美组合。在过去的十几年里,基于不同CE-MS分离模式的高性能手性分析体系层出不穷,如电动色谱-质谱(EKC-MS)、胶束电动色谱-质谱(MEKC-MS)和毛细管电色谱-质谱(CEC-MS)等,并成功应用于医药、生物、食品和环境科学等领域的手性化合物分析。该文主要综述了2011~2021年,CE-MS在手性化合物分析领域的技术、手性选择剂(如改性环糊精和聚合物表面活性剂等)的使用以及在医药等领域应用方面的研究进展,并讨论了不同手性分析模式的局限性,为未来的CE-MS手性分离分析技术发展及应用提供借鉴。

## Abstract (en)

Most drugs used to treat diseases are chiral compounds. Drug enantiomers possess similar physical and chemical properties but may feature distinct pharmacological activities. Drug enantiomers may also exhibit different or even opposite functionalities for metabolism, in terms of the metabolic rate and toxicity in the body. Therefore, it is imperative to analyze, separate, and purify the enantiomers of drugs. The separation of chiral compounds is essential for drug research and development. It is also of significance in various fields including biological environments, food, and medicine.

Various highly selective and sensitive methods have been developed for the quantitative and qualitative analyses of chiral compounds. A typically employed technique is high performance liquid chromatography-mass spectrometry (HPLC-MS). While HPLC-MS offers high sensitivity and reproducibility, it requires expensive chiral columns and MS-compatible mobile phases for the chromatographic column. Further, the column efficiency and resolution capacity in chiral chromatography packing require improvement. Recent progress has shown that capillary electrophoresis-mass spectrometry (CE-MS) has broad applications in chiral analysis. As a well-established analytical technique, CE-MS combines the highly efficient separation technique of CE with the highly sensitive detection technique of MS. Thus, it offers many essential advantages for analysis. For example, CE-MS has a high separation efficiency and requires very low amounts of samples and reagents. It can also achieve sensitive and selective determination, and the obtained diversified separation modes can be used for different samples. Therefore, CE-MS has proved to be important in analytical chemistry, especially in proteomics and metabolomics.

CE can also exhibit excellent performance in chiral separation. Hence, combined with the sensitive detection technique of MS, CE-MS would be ideal for chiral analysis. Chiral CE-MS can provide a wide range of qualitative information on samples simultaneously in a single run, including the migration time, relative molecular mass, and ionic fragments. It addresses the challenges associated with identifying unknown chiral compounds in actual samples (including chiral compounds without UV absorption groups or fluorescence groups). The high-throughput analysis of multiple groups of chiral enantiomers can be achieved while mitigating the matrix effect of biological samples. In the last ten years, high performance chiral analysis strategies based on different CE-MS modes have been developed. These include electrokinetic chromatography-mass spectrometry (EKC-MS), micellar electrokinetic chromatography-mass spectrometry (MEKC-MS), and capillary electrochromatography-mass spectrometry (CEC-MS). CE-MS has been successfully applied in chiral analysis in various fields such as medicine, biology, food, and environmental science.

CE-MS is promising in the chiral analysis of drugs, especially for drug development and drug quality control, as well as pharmacokinetics and pharmacodynamics research. Recent studies have focused on the development of MS-friendly and highly selective chiral analytical methods, which will broaden the application of CE-MS. In CEC-MS chiral analysis, more attention has been paid to developing novel capillary chiral stationary phases for monolithic or packed columns. Because of the diversity of chiral selectors for EKC-MS and MEKC-MS, the chiral analysis of drugs using these techniques has attracted intense research interest. Moreover, functional nanoparticles have been employed to increase the surface area of the CEC columns for enhancing the efficiency of chiral analysis. The chiral separation and analysis of miniaturized microchip equipment via CE-MS has also been explored, but remains to be widely used in practical applications.

The purpose of this review is to provide insights that would aid in broadening the applications of CE-MS to chiral analysis. In this review, we primarily summarize research progress on the application of CE-MS to chiral analysis, based on the literature published during the years 2011-2021. Chiral selectors (e. g., modified cyclodextrin and polymer surfactants) and their reported applications in CE-MS are presented. The determination results for drug enantiomers using different CE-MS modes are compared. The application of CE-MS in other research fields is also presented, along with the advantages and limitations of different CE-MS methods.

手性是自然界和生命体的基本属性之一,诸如生物结构中的核酸、蛋白质及糖类等都具有手性^[1⇓-3]^。目前绝大多数药物都是以手性形式存在,这些药物在生命体内的药理活性、代谢作用和速率及毒性等方面均存在显著差异,比如一种对映体有活性,而另一种无显著的药理活性,甚至有毒副作用或可发生拮抗作用^[4]^。除了旋光性上的差异,手性药物具有相同的物理和化学性质,故对其分离分析一直都是药物分析、分离纯化领域研究的重点和难点。新药的研发和应用亦需要研究人员继续开发新的高效手性分析方法,以实现高选择性和高灵敏度的手性化合物定量和定性分析。

高效液相色谱-质谱(HPLC-MS)具有较高的灵敏度和重现性,是目前手性药物分离分析的主要方法^[5⇓-7]^。然而,HPLC-MS需要昂贵的手性柱^[8]^和与MS兼容的色谱柱流动相,而且手性色谱填料的柱效和拆分能力仍有待提高。毛细管电泳(CE)技术凭借其高效、低样品消耗、分析快速、分离模式多样化等诸多优势^[9⇓-11]^,已经发展成为手性分离研究领域极具吸引力和应用前景的分析方法之一^[12⇓-14]^。紫外可见检测器(UV-Vis)是CE最常用的检测器,但是毛细管的光程长度较短,导致灵敏度较低,因此难以满足生物样品中痕量手性化合物的分析要求。激光诱导荧光检测器(LIF)可以提高检测的灵敏度,但是只适用于本身带有荧光或被荧光标记的物质。而毛细管电泳-质谱联用技术结合了CE的分离效率高、分析速度快、样品消耗低以及MS的高灵敏度和强结构解析能力,近些年来在蛋白质组学和代谢组学等领域发挥了重要作用^[15⇓⇓-18]^。CE杰出的手性拆分能力与MS优势的结合,亦使CE-MS成为实现手性化合物高效分离分析的完美组合,尤其是在复杂生物基质中手性化合物分析的灵敏度和分辨率方面,为药物、医学以及食品科学等领域重要手性分子分析提供了新视角^[19⇓-21]^。手性CE-MS联用技术,在一次分析中能同时得到样品的迁移时间、相对分子质量和离子碎片等定性信息,解决了实际样品中未知手性化合物(包括无紫外吸收基团或荧光基团的手性化合物)的识别问题,在减少生物样品基质效应的同时,可以对多组手性对映体实现高通量分析^[22⇓-24]^。

在过去的十几年里,基于不同CE-MS分离模式的高性能手性分析体系层出不穷,并成功应用于医药、生物、食品和环境科学等领域的手性化合物分析中^[25⇓⇓-28]^。这篇综述着重评述了电动色谱-质谱(EKC-MS)、胶束电动色谱(MEKC-MS)和毛细管电色谱-质谱(CEC-MS)手性分离模式从2011年到2021年的最新发展和应用。综述介绍了CE-MS各种手性分析模式下的分离原理、手性选择剂以及在医药等领域中重要手性化合物的分析应用,并讨论了不同手性分析模式的局限性。最后总结了CE-MS联用模式在手性化合物分离分析中的应用前景。

## 1 CE-MS手性分析模式

用于手性分析的CE-MS主要模式有EKC-MS、MEKC-MS和CEC-MS。

EKC-MS模式通过向挥发性缓冲溶液中添加极低浓度的手性选择剂来实现对映体分离。手性选择剂(如*α-*、*β-*或*γ*-环糊精(CDs)及其衍生物、冠醚、大环抗生素与蛋白质等)作为准固定相(PSP),在EKC分离过程中与中性和带电的手性分析物相互作用,由MS分析检测。两种对映体之间没有电泳迁移率的差异,因此无法实现对映体的分离。而对映体与手性选择剂相互作用的不同,导致其表观有效迁移率的差异,从而实现对映体分离。EKC-MS具有较高的分离效率,即使手性选择剂与游离手性分析物结合的差异很微小,也会使对映体分离。当使用表面活性剂或聚合物/分子胶束时,这种EKC-MS模式称为MEKC-MS。在MEKC-MS中,将未聚合的胶束或形成分子胶束的可聚合带电表面活性剂添加到挥发性缓冲液中以实现目标物的手性分离。CEC-MS模式中,对映体的分离是在固定手性固定相的毛细管柱中实现的。一方面,CEC结合了HPLC高选择性和CE的高分离效率,避免了非挥发性手性选择剂进入MS检测器而导致离子抑制和离子源污染。另一方面,MS作为CE检测器,不仅提供了高灵敏的分析,而且还提供了丰富的结构信息。

EKC-MS、MEKC-MS和CEC-MS模式的分离原理都是基于手性混合物中化合物与手性固定相或手性选择剂相互作用的差异。目前,CEC-MS的研究重点集中于开发和制备新型高效修饰手性柱的方法,而EKC-MS和MEKC-MS的研究工作更多关注开发新的应用领域。此外,对于手性化合物,可通过在手性中心的不同位置添加衍生部分,对映体衍生化后形成非对映体,衍生物的电泳迁移率不同,从而通过常规毛细管区带电泳实现分离分析。

## 2 EKC-MS

### 2.1 EKC-MS手性分析原理

20世纪末,Schulte等^[29]^首次提出EKC-MS分离模式用于手性化合物的分离。作为CE-MS的重要分离模式之一,EKC-MS分离模式通过在挥发性缓冲溶液中直接加入手性选择剂,实现了手性分离。手性选择剂(又称准手性固定相)与手性化合物之间包合、疏水、氢键、静电和范德华力等相互作用的差异导致对映体电泳迁移率的不同,从而实现手性化合物的分离。EKC-MS最常采用的手性选择剂是非挥发性的环糊精(如*α-*、*β-*、*γ*-CDs及其衍生物)^[30]^。若非挥发性化合物进入MS,可能产生离子抑制和离子源污染,因此导致MS分析灵敏度的降低。反向迁移技术(CMT)和部分填充技术(PFT)的应用可以避免不兼容的手性选择剂进入离子源,图1为基于CMT和PFT的手性EKC-MS原理图。CMT适用于使用带电手性选择剂(非中性)的手性分析。

**图1:**
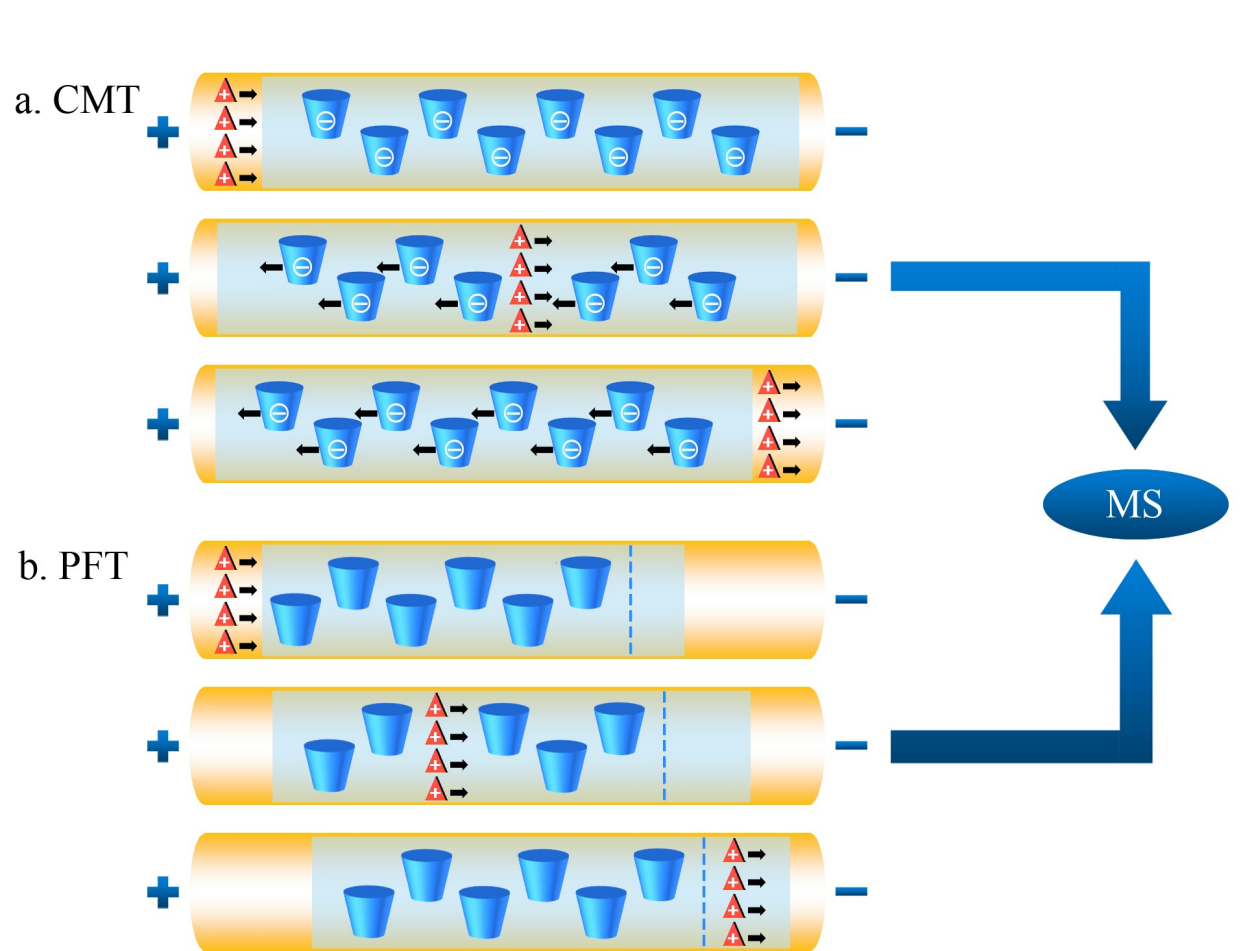
(a)使用带负电荷CD的CMT和(b)使用中性CD的PFT检测阳离子手性化合物的示意图

图1a中使用的手性选择剂为带负电荷的CD,利用CMT分析阳离子手性对映体。阳离子化合物在电场作用下向MS方向迁移,而带负电荷的CD向相反方向迁移,也就是远离MS的方向。图1b使用电中性CD作为手性选择剂,利用PFT分析阳离子手性化合物,首先用不含有手性选择剂的缓冲溶液充满毛细管,然后在毛细管的入口端引入一段含有手性选择剂的缓冲溶液(以避免手性选择剂进入离子源),在电场作用下,手性化合物经过含有手性选择剂的缓冲溶液后实现分离。最终,分离得到的对映体进入不含有手性选择剂的电泳缓冲液中并向MS方向迁移,而电中性CD的迁移速度远小于阳离子手性化合物且只在毛细管的入口端引入一段,因此避免了CD对离子源的污染。虽然CMT和PFT方法在EKC-MS中经常使用,但与传统的EKC或MEKC方法相比,它们将含有手性选择剂的手性CE与不含手性选择剂的非手性CE相结合,且具有较短的分离长度,因此存在分辨率低、选择性不同以及峰容量较低等缺点。

### 2.2 EKC-MS的准手性固定相及应用

Tanaka等^[31]^于2000年首次提出将冠醚作为手性选择剂,结合PFT方法的手性EKC-MS,实现了外消旋氨基吡咯烷、氨基己内酰胺和环丝氨酸的对映体分析。手性EKC-MS通过结合PFT来避免质谱检测器的信号抑制和污染,实现了手性化合物灵敏稳定的分离分析。Rollman等^[32]^采用0.125%高硫酸盐-*γ*-环糊精(HS-*γ*-CD)和15 mmol/L (+)-18-冠醚-6-四羧酸((+)-18-C-6-TCA)相结合作为手性选择剂,使用在线无鞘流的EKC-MS实现了8种卡西酮衍生物及其位置和光学异构体的分离。其中,在分离戊酮对映体时,PFT与EKC-MS技术(图2a)的联用与不采用PFT方法(图2b)相比,其分离效率从27000增至140000,分离度从5.1增至9.6。

**图2:**
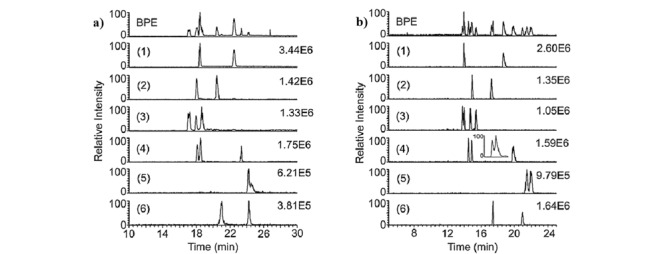
分析物分离度和强度的(a)基本峰电泳谱图(BPE)和(b)提取离子流电泳谱图^[32]^

Chen等^[33]^以2-羟甲基-*β*-环糊精作为手性选择剂,结合PFT方法的手性EKC-MS技术实现了植物性药物的手性选择性分析。这种方法可以直接测定复杂基质中的分析物,无需任何预处理步骤,而且灵敏度高,操作成本低。Na等^[34]^采用羧基冠醚(18C_6_H_4_)作为手性选择剂,结合PFT方法的手性EKC-MS技术对未衍生化氨基酸对映体(AAs)进行了手性拆分,17对氨基酸的理论塔板数(*N*)为3.3×10^4^~11.8×10^4^,分离度(*R*_s_)为0.5~21,其中12对氨基酸得到基线分离。2021年,Benavente等^[35]^以0.5%硫酸-*α*-CD为手性选择剂,10 mmol/L pH 7的醋酸铵为缓冲溶液,采用基于PFT的高压在线固相萃取毛细管电泳-质谱联用法测定尿液中*R*,*S*-3,4-亚甲基二氧吡喃缬草酮(*R*,*S*-MDPV)对映体的含量。该方法测得的*R*,*S*-MDPV对映体的线性范围为30~250 ng/mL, LOD为10 ng/mL, RSD均小于10.5%。

尽管开发的PFT、CMT方法和开管柱、填充柱、整体柱可以解决CE-MS技术在手性分离检测中离子源污染的问题,以及手性选择剂(通常是非挥发性CD及其衍生物)在背景电解质(BGE)中引起的电离抑制,然而这些方法往往会降低手性分辨率、峰值容量和稳定性。实际上,考虑到CE缓冲液和鞘流液的稀释作用,能够引入质谱仪的非挥发性手性选择剂的绝对量非常低。因此,在CE-MS系统中直接引入低浓度手性选择剂一定程度上也可以减少离子源污染和电离抑制,并获得足够的灵敏度。2019年,Liu等^[36]^建立了一种CE-MS方法来实现大鼠脑脊液中具有2个手性中心的3-羟基天冬氨酸的4种立体异构体(L-苏-3-羟基天冬氨酸酯(2*S*,3*S*,L-THA)、D-苏-3-羟基天冬氨酸酯(2*R*,3*R*,D-THA)、L-赤-3-羟基天冬氨酸酯(2*S*,3*R*,L-EHA)、D-赤-3-羟基天冬氨酸酯(2*R*,3*S*,D-EHA))的同时分离和鉴定。氯甲酸-9-芴甲酯(FMOC-Cl)作为衍生化试剂辅助3-羟基天冬氨酸对映体的分离,该衍生化试剂的使用减少了分析物的迁移时间和*β*-CD的用量,不仅有利于MS的检测而且还进一步提高了分析的灵敏度。在最佳实验条件下,使用CE-MS成功实现了具有2个手性中心的3-羟基天冬氨酸的4种立体异构体的同时分离分析,迁移时间和峰面积的RSD分别低于1.43%和2.56%, 4种立体异构体在加标大鼠脑脊液中的回收率为91.2%~99.5%。该方法成功应用于大鼠脑脊液中D-EHA的分析。

Sebestova等^[37]^采用CE-电感耦合等离子体(ICP)-MS技术实现了奥沙利铂对映体的分离分析,与CE-ESI-MS技术相比,该方法具有独特的优势,即允许使用“标准”的电解质(含有非挥发性运行电解质成分和添加剂,如手性选择剂等)。CE-ICP-MS技术由Olesik等^[38]^于1995年提出,目前主要用于形态分析和金属与配体相互作用的研究,包括金属基纳米颗粒的研究^[39]^。这一方法解决了手性EKC与MS联用时,非挥发性的手性选择剂可能会进入MS引起的离子抑制和离子源污染,从而降低灵敏度的问题。实验结果表明,CE-ICP-MS技术动态范围广(0.1~500 μg/mL),灵敏度高,可实现约49 fg(125 amol)的奥沙利铂对映体的检测,并成功用于尿样中奥沙利铂对映体的分析。

## 3 MEKC-MS

### 3.1 MEKC-MS手性分析原理

手性MEKC技术由Terabe等^[40]^首次提出。该方法通过在缓冲溶液中添加浓度高于临界胶束浓度的手性表面活性剂,使其形成手性分子胶束相,由此可以根据手性化合物在胶束相与水相之间分配系数的差异来实现手性分离。MEKC-MS的手性分离原理与EKC-MS的类似,区别在于前者进行手性化合物的分离时采用的是表面活性剂或聚合物分子胶束作为准手性固定相^[41]^。图3为采用阴离子聚合物分子胶束作为准手性固定相的MEKC-MS分离机理。与带电荷和较小相对分子质量的手性选择剂相比,手性分子胶束(MoM)在溶液中是携带多个负电荷的聚合物,其电泳有效迁移速度非常大且迁移方向是从阴极到阳极,不易流向阴极MS端。若电渗流(EOF)的方向是从阳极到阴极,当EOF的迁移速度(*μ*_EOF_)大于MoM的迁移速度(*μ*_MoM_)时,手性MoM最终会在一定的时间(*t*_MoM_)内洗脱出来。当*μ*_EOF_小于*μ*_MoM _时,手性MoM将不会被洗脱出来,*t*_MoM _是无限的。亲水的极性和中性手性化合物(图3中的实心五角星)仅在手性分子胶束表面发生偶极-偶极相互作用,因此手性化合物会被分子胶束弱保留,而与EOF共同洗脱(甚至有些时候比EOF先洗脱),迁移速度为*μ*_a_。相反,疏水的非极性手性化合物(图3中的实心三角形)将被吸附到分子胶束的核心,并在*t*_MoM_或*t*_MoM_附近洗脱,迁移速度为*μ*_c_。极性在两极之间的中极性手性化合物(图3中的实心正方形)将基于静电、疏水和氢键与分子胶束之间相互作用力的强弱进行洗脱,并在胶束洗脱窗口内实现分离,迁移速度为*μ*_b_。

**图3:**
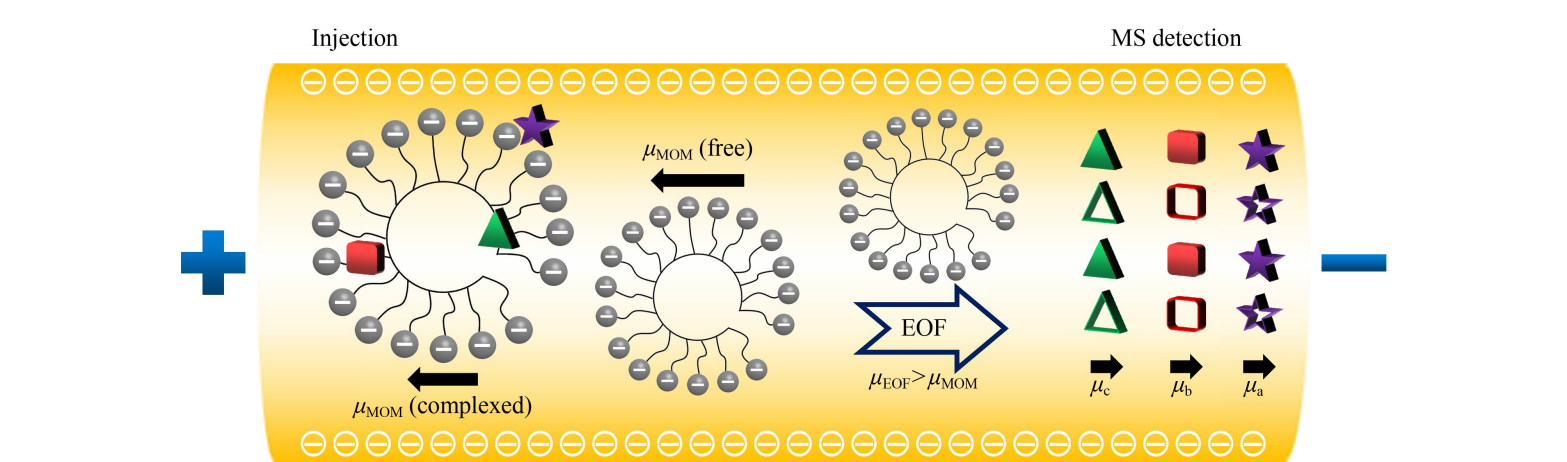
阴离子聚合物表面活性剂在手性MEKC中的分离机理

基于手性分子胶束的MEKC方法已经广泛用于化合物的手性分离,但当与MS联用时,其应用仍具有局限性。比如,为了提高对映体选择性,需要使用高浓度的表面活性剂形成胶束,但高浓度胶束往往会抑制ESI-MS信号和导致离子源喷雾室的污染。此外,在运行缓冲液中通常使用的较高浓度有机溶剂(用来提高疏水性手性化合物的溶解度)会破坏胶束的形成,从而影响疏水溶质的手性识别,缩小疏水性范围宽的多种手性对映体(比如原药及其代谢产物对映体)的分析窗口。因此,当手性MEKC与MS联用时,往往选择临界浓度为零的相对分子质量较高的MoM。

### 3.2 MEKC-MS的分子胶束及应用

Shamsi等^[42]^首次提出将手性聚合物表面活性剂用于MEKC-MS中,实现手性化合物的分离。聚合物表面活性剂(分子胶束、MoM)具有多种手性官能团和链长,通常在手性MEKC-MS中作为准手性固定相,是最具潜力的手性选择剂之一,开发具有高分离选择性和高MS兼容性的手性MoM亦成为研究热点。

2016年,Shamsi等^[43]^合成了一种新型*N*-烷基烯基*α*-D-吡喃葡萄糖苷聚钠表面活性剂(poly-*α*-D-SUGP),用于MEKC-MS分离不同的手性药物。在最佳MS喷雾室条件下(30 kV和25 ℃),以20 mmol/L NH_4_OAc和25 mmol/L poly-*α*-D-SUGP为BGE,使用手性MEKC-MS在最佳pH为5.0时,实现了麻黄碱生物碱对映体的手性分离,在pH为7.0时,实现了*β*-受体阻滞剂两类阳离子药物对映体的手性分离(见图4),检出限分别为10 ng/mL和50 ng/mL。2016年,Liu等^[44]^用所合成的吡喃葡萄糖基作为MoM,采用手性MEKC-MS/MS技术对多个手性化合物进行高通量筛选,实验结果表明所合成的吡喃葡萄糖基与电喷雾电离MS完全兼容,可以作为手性选择剂。

**图4:**
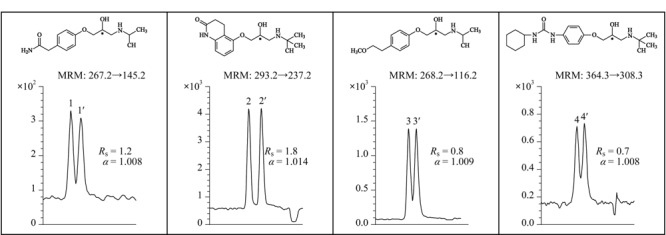
MEKC-MS/MS分析技术用于*β*-受体阻滞剂对映体分离的电泳谱图^[43]^

基于缓冲溶液中添加手性MoM的MEKC-MS联用技术,成功应用于手性化合物的分离,但仍然存在保留时间和峰面积重现性差的问题,而且很难控制未修饰毛细管的电渗流。为了解决这一问题,Shamsi等^[45]^于2020年提出有效策略,在MEKC-MS方法中采用共价键合2-丙烯酰胺-2-甲基-1-丙磺酸(AMPS)的色谱柱,并在缓冲溶液中添加聚钠*N*-十一烯氧羰基-L-亮氨酸(poly-L-SUCL)作为MoM,在25 min内实现了3种*β*阻滞剂阿替洛尔(ATEN)、美托洛尔(METO)和品多洛尔(PINDO)对映体的同时分离和MS/MS检测,柱寿命至少提高了45~50次,检出限低至0.2 μg/mL。

为了提高分析通量并降低分析成本,Fillet等^[46]^建立了全自动在线衍生-MEKC-MS方法,以(-)-1-(9-芴基)氯甲酸乙酯(FLEC)为标记试剂,进行D-和L-氨基酸的全自动手性分析。在最佳的实验条件下(样品和FLEC区带的比例为2∶1, BGE为150 mmol/L全氟辛酸铵(APFO), pH=9.5),实现了8种氨基酸的完全手性拆分和6种氨基酸的部分手性拆分。实验结果表明,该方法在痕量浓度范围内仍然具有良好的重复性,并通过对人工脑脊液样品的分析,验证了该方法对生物样品分析的适用性。

## 4 CEC-MS

CEC-MS是一种兼具CEC高效分离以及MS高选择性和高灵敏度的新型分离分析技术,因其在生物及药物分析中具有非常独特的分离与检测优势而倍受关注,也为生命科学、药物和环境等研究领域手性化合物的定量、定性分析提供了强有力的工具。手性CEC是在毛细管柱中填充或在管壁涂布、键合手性固定相,在高压电场作用下,流动相在电渗流的驱动下通过手性毛细管柱。对于手性对映体,其手性分离主要通过在手性固定相和流动相之间的分配差异实现。根据手性选择剂在毛细管柱中的固定形式,CEC可以分为3种类型:填充柱CEC、整体柱CEC和开管柱CEC。近十年,开管柱CEC-MS手性分离研究和应用鲜有报道,这可能是由于其柱效和柱容量低,导致分离性能受限。目前更多的研究工作集中在填充柱和整体柱CEC-MS毛细管手性柱的开发和应用上。

### 4.1 填充柱CEC-MS

#### 4.1.1 填充柱CEC-MS的手性分析原理

作为一种CEC常用分离模式,填充柱CEC于1993年被Li等^[47]^首次提出,色谱柱制备是将色谱填料填充到毛细管中作为固定相。毛细管两端通常通过烧结制备柱塞将填料固定在毛细管内,其主要缺点是柱的制备繁琐困难,而且柱塞容易使毛细管内产生气泡,导致保留时间不重现,如果产生的气泡过多甚至会阻断电流。在填充柱CEC-UV分析中,可以通过在毛细管的两端加压以减少气泡的产生,但是这种方法不适用于填充柱CEC-MS的分析,这是由于MS检测需要填充柱毛细管的出口端保持在大气压下。针对这些问题,在手性填充柱CEC中,使用一端拉伸为锥形的单堵头毛细管柱可以克服以上缺点。将手性选择剂修饰在二氧化硅载体表面并填充于毛细管柱内,通过与手性填料形成的氢键以及分子间的相互作用,可实现对映体的有效分离(见图5a)。大多数CEC方法都采用与MS方向一致的强EOF,因此锥形端被放置在与MS相连的毛细管出口端。此外,锥形填充柱CEC可以提供更好的对映体选择性和更高的样品装载能力,在手性化合物分离领域有很好的应用价值。

**图5:**
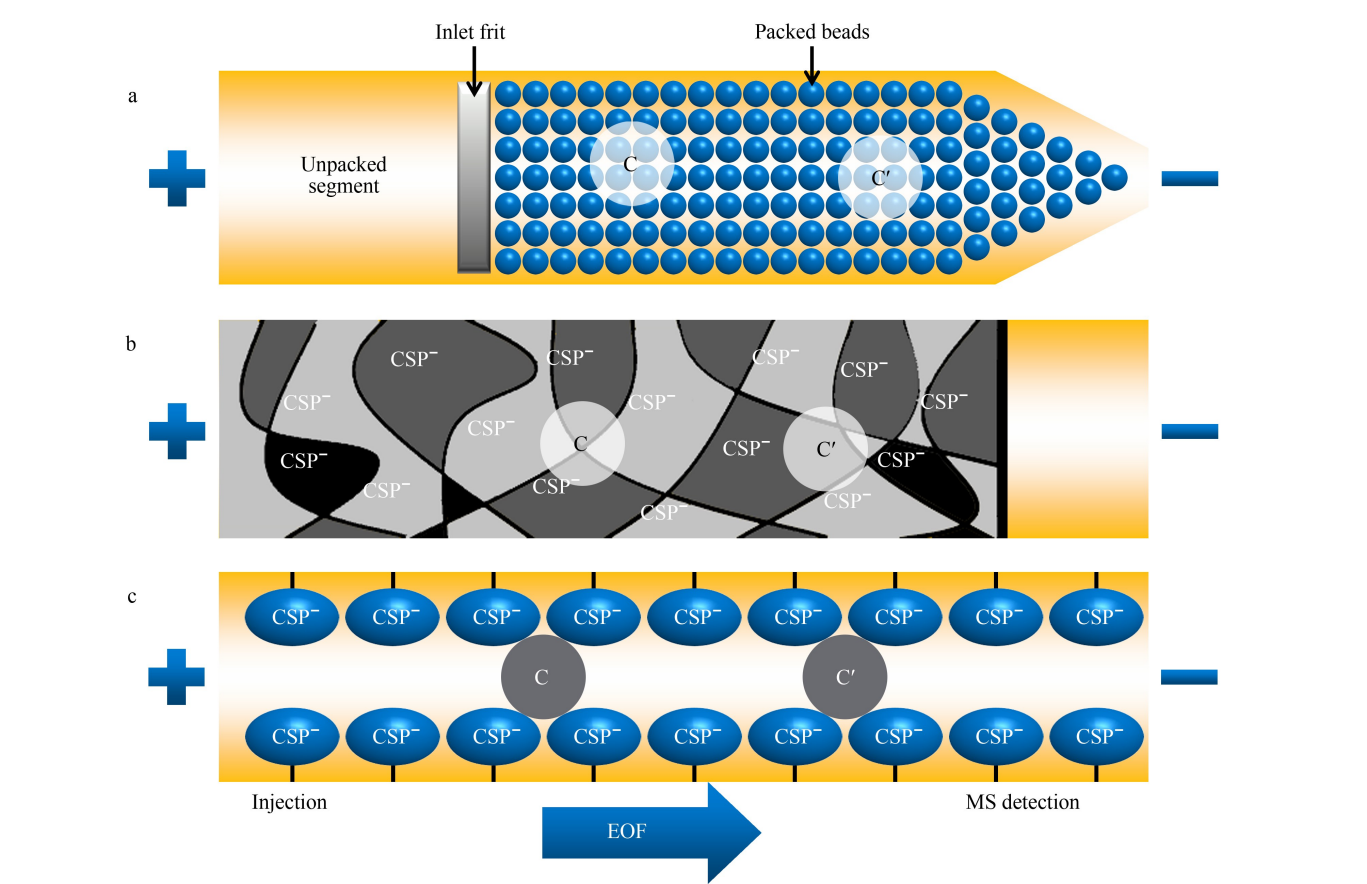
(a)填充柱、(b)整体柱和(c)开管柱手性CEC的分离机理

#### 4.1.2 填充柱CEC-MS的手性固定相及应用

传统CEC填充柱的制备通常需要双堵头或单堵头来固定颗粒基手性固定相(CSP)填充材料,然而这种方法对于CEC-MS的检测仍然存在若干缺陷。近几年,无堵头填充柱手性CEC-MS的研究引起了人们的广泛关注。2011年,Bragg等^[48]^首次提出了无堵头填充柱手性CEC-MS,使用一种新型的双通连接器(PicoClear型,New Objective公司,美国)将两根内锥形毛细管填充柱连接在一起,制备了应用于CEC-MS的无堵头填充柱。作者通过对氨鲁米特对映体的手性分离分析,验证了该无堵头填充柱的稳定性与可行性。比较无堵头填充柱CEC-MS与单堵头填充柱CEC-MS的精密度,连续运行90次测得的日间RSD分别为3.7%和5.5%。实验结果表明,无堵头填充柱CEC-MS具有较高的稳定性与可靠性。使用相同的手性固定相时,无堵头填充柱CEC-MS比单堵头填充柱CEC-MS具有相似的灵敏度和分离度,但是分离效率更高。

对于填充柱CEC-MS的手性分析,分析时间较长仍是手性化合物分析的瓶颈。最近,Bragg等^[49]^使用仅有7 cm长的单堵头填充柱用于手性化合物的CEC-MS高通量分析。比较了两种纤维素基填充柱,分别使用三(3,5-二甲基苯基氨基甲酸酯)纤维素(CDMPC)和磺化三(3,5-二甲基苯基氨基甲酸酯)纤维素(CDMPC-SO_3_)作为手性固定相用于CEC-MS对映体的快速分离。与中性手性固定相(如CDMPC)相比,带电荷的手性固定相(如CDMPC-SO_3_)可以在更短时间内实现谷酮酰亚胺、氨基谷酮酰亚胺、华法林和2,2,2-三氟-1-(9-蒽基)乙醇对映体的基线分离。该研究为使用短手性填充柱进行高通量手性分析提供了可能。

### 4.2 整体柱CEC-MS

#### 4.2.1 整体柱CEC-MS的手性分析原理

Schweitz等^[50]^于1997年首次提出整体柱CEC用于手性分离,通过有机或无机的方法在无需柱塞的情况下,在毛细管内原位固化或原位聚合多种功能基团,制备具有多孔层结构的整体式固定相。手性整体柱具有制备方法简单、比表面积大、柱效高等优点。如图5b所示,在整体柱CEC中,阴离子CSP形成多孔聚合物并原位固定在毛细管壁上。CSP的阴离子交联单体产生EOF后,基于CSP与手性化合物之间的静电、离子配对和疏水作用的结合,将手性化合物拆分。与填充柱CEC-MS相比,整体柱手性CEC是聚合物形成的渗透性和多孔性较好的材料棒,具有锥形填充柱手性CEC的优点,且无需堵头,不容易形成气泡,因此手性整体柱CEC与MS联用得到了广泛关注和研究。

#### 4.2.2 整体柱CEC-MS的手性固定相及应用

整体柱手性CEC-MS中,整体柱是聚合物形成的单一连续多孔材料棒,无需末端柱塞。与颗粒填充柱相比,手性整体柱具有丰富的介孔。*β*-CD及其衍生物(*β*-CDs)具有一定尺寸的立体手性空腔,可以与许多有机无机分子、离子等通过范德华力、静电引力、疏水作用形成主-客体包合物,具备分子识别和选择性结合的能力,是一种常用的色谱手性固定相,在手性拆分领域应用广泛。通过对*β*-CD改性不但可以增加其对手性化合物的拆分能力,还可以将其应用在CEC中,作为整体柱的手性固定相。

2017年,高立娣等^[51]^以自制的顺丁烯二酸酐-*β*-环糊精(MAH-*β*-CD)为手性选择剂制备CEC整体柱,结合电喷雾-飞行时间/质谱技术(ESI-TOF/MS)用于分离检测两种*β*_2_-受体激动剂药物盐酸克伦特罗(Cle)和盐酸班布特罗(Bam)。使用整体柱CEC-ESI-TOF/MS技术可以实现两种*β*_2_-受体激动剂药物Cle和Bam的基线分离,分离度分别为2.31和1.83,为该类药物的手性分离提供了一种新方法。2018年,高立娣等^[52]^又提出以乙烯基的*β*-环糊精(UPA-*β*-CD)为功能单体,乙二醇二甲基丙烯酸酯(EDMA)为交联剂,运用原位聚合法制备了新型CEC整体柱。在手性整体柱CEC-MS模式下成功拆分了盐酸奥昔布宁、盐酸苄丝肼两种手性药物,其分离度分别为1.76和2.12。随后,高立娣等^[53]^于2019年以烯丙基咪唑鎓-*β*-环糊精(AI-*β*-CD)毛细管电色谱整体柱作为手性分离柱,以D,L-酪氨酸为分析物对整体柱CEC-ESI-TOF/MS技术进行性能评价,并对手性除草剂甲氧咪草烟对映体进行分离,分离度为2.25。后续,通过优化CEC分离条件(缓冲溶液pH=8.5,分离电压15 kV和分离温度20 ℃)和MS检测条件(鞘流液的流速为0.6 mL/min),采用整体柱CEC-ESI-TOF/MS技术对3种混合氨基酸对映体(D,L-精氨酸、D,L-缬氨酸、D,L-谷氨酸)进行手性分离,15 min内实现了3种混合氨基酸对映体6个组分的分离,分离度分别为3.03、1.59和1.37(见图6)^[54]^。最近,高立娣等^[55]^同样利用实验室自制MAH-*β*-CD作为手性固定相,制备了MAH-*β*-CD电色谱整体柱,采用CEC-ES-TOF/MS对盐酸地尔硫卓和盐酸维拉帕米混合手性药物进行分离检测,获得了满意的结果。

**图6:**
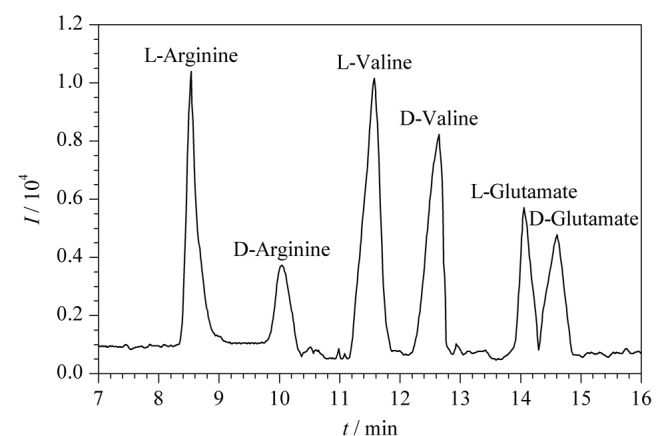
混合氨基酸对映体的总离子流色谱图^[54]^

### 4.3 开管柱CEC-MS

#### 4.3.1 开管柱CEC-MS的手性分析原理

1982年,Tsuda等^[56]^首次提出了开管柱CEC,通过在毛细管内壁键合、涂覆或吸附单层或多层含有带电或带有手性选择剂官能团的色谱固定相,以制备空心式开管毛细管分离柱,实现手性化合物的分离分析。原理如图5c所示,CSP通过键合、涂覆或吸附作用固定在毛细管内壁,基于两种对映体(C和C')在流动相和CSP之间的分布差异而实现对手性化合物的分离。与填充柱CEC相比,开管柱CEC无需柱塞和填料,制备过程简单,不容易产生气泡和涡流扩散效应,具有表面改性和固定化化学的多样性和实用性等特点。

#### 4.3.2 开管柱CEC-MS的手性固定相及应用

虽然开管柱CEC避免了颗粒填充过程和柱塞制作,毛细管内壁修饰过程简单省时,但是其柱效和柱容量均低于填充柱CEC。而且当开管柱CEC与MS联用检测时,如果熔融石英毛细管内壁涂层修饰不稳定,脱落的涂层修饰有可能污染MS离子源。因此,近几年开管柱CEC与MS联用技术用于手性化合物的分离研究报道较少。2015年,高立娣等^[57]^采用开管柱CEC-ESI-TOF/MS联用技术分离分析盐酸异丙肾上腺素和盐酸氯丙那林混合手性药物。利用实验室自制的有机-无机杂化开管柱作为色谱分离柱,在最佳的分离检测条件下(NH_4_Ac-HCOOH缓冲溶液的浓度20 mmol/L、pH值4.0、运行电压20 kV、分离温度20 ℃、鞘液的组成为含5 mmol/L NH_4_Ac的50%甲醇水溶液、鞘液的流速0.4 mL/min),18.5 min内实现了2种混合手性药物4个组分的基线分离,4个组分的分离度分别为1.62、4.68和1.53(见图7)。实验结果表明,该方法的分离分析效率高,试剂消耗量少,运行成本低,对手性药物联合用药后的残留及其他手性药物的分离检测具有一定参考价值。

**图7:**
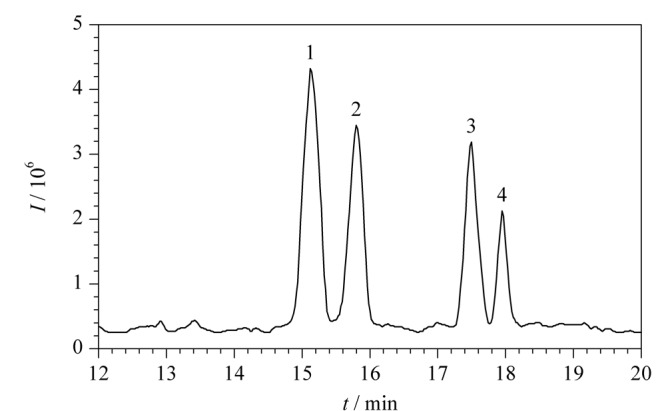
盐酸氯丙那林和盐酸异丙肾上腺素混合物的总离子流图^[57]^

## 5 总结和展望

相比于广泛应用的HPLC-MS, CE-MS凭借其高效率、低消耗、高选择性、分离模式多样化等诸多优势,已发展成为手性分析领域应用前景广阔的分析方法之一,并且已成为HPLC-MS等其他经典手性分离方法的一个强有力补充技术。

目前CE-MS手性分析的研究挑战之一是实现快速和超灵敏的手性分析。采用基于短毛细管的快速毛细管电泳(HPCE)结合在线样品富集有望解决这个难题。此外,CE-MS的不同手性分析模式大多数采用的是三管设计的鞘状流动界面,灵敏度较低。新进研发的新型界面技术,如通过微瓶辅助的界面流动^[58]^、无套多孔尖端的设计^[32]^以及CE-MS离子源的引入^[59]^等,在提高手性化合物分析灵敏度方面显示出巨大应用前景。另一方面,开发同时对多种手性药物进行对映体分离、检测和定量的CE-MS手性分析方法,也是目前研究的重点和难点。这些研究将对开发制药工业中的通用方法和高通量分析生物样品中的手性药物及其手性代谢物具有重要意义,对手性药物和代谢物的药物-药物相互作用和毒性研究也具有指导价值。EKC-MS和MEKC-MS应用中的手性选择剂具有多样性,使其在新药开发和药物质量控制、药代动力学以及药效学研究中具有巨大的潜力。进一步开发MS友好、绿色和高选择性的手性选择将拓宽待分离手性化合物的应用范围。目前,CEC-MS手性分析研究中,研究者更多致力于开发用于整体柱或填充柱的新型毛细管手性固定相。使用功能化纳米颗粒增加CEC手性柱表面积以及CE-MS的微型化微芯片设备的研发,目前仍是尚未充分探索的领域,尤其在实际应用方面与相对更加通用的手性分离模式相比仍有较大差距。
